# Steric Determinants of Pt/DNA Interactions and Anticancer Activity

**DOI:** 10.1155/MBD.1998.197

**Published:** 1998

**Authors:** Trevor W. Hambley, Susan J. Berners-Price, Murray S. Davies, Connie I. Diakos, Hui Meng Er, Ronald R. Fenton, Edwina C. H. Ling, Evonne M. Rezler

**Affiliations:** 1 School of Chemistry University of Sydney NSW 2006 Australia; 2 School of Science Griffith University Nathan QLD 4111 Australia

## Abstract

Studies directed at establishing the structural features that control Pt/DNA interactions and the anticancer activity of Pt drugs are described. [^1^H, ^15^N]-HSQC 2D NMR spectroscopic studies of the reactions of cisplatin with oligonucleotides containing ApG and GpA binding sites reveal dramatic differences in the rates of formation of monofunctional adducts at the two sites. When the reactant is *cis*-[Pt(NH^3^)^2^(OH^2^)^2^]^2+^ no such differences are observed suggesting that outer-sphere interactions between the reactant and the oligonucleotide may play a substantial role in determining the rates. Rates of closure to the bifunctional adducts are similar to those observed for cisplatin. Studies of the adduct profiles formed by sterically bulky and/or optically active complexes reveal that steric interactions play a major role in mediating the binding of Pt(ll) to DNA but that hydrogen bonds play less of a role. *In vitro* cytotoxic activities for these complexes do not always follow the trends that would be expected on the basis of the adduct profiles.


### STERIC DETERMINANTS OF PT/DNA INTERACTIONS AND ANTICANCER ACTIVITY

Trevor W. Hambley*[1], Susan J. Berners-Price[2], Murray S. Davies[1], Connie I. Diakos[1], Hui Meng Er[1], Ronald R. Fenton,[1] Edwina C.H. Ling[1] and Evonne M. Rezler[1]

[1] School of Chemistry, University of Sydney, NSW 2006, Australia
[2] School of Science, Griffith University, Nathan QLD 4111, Australia

**Abstract**
Studies directed at establishing the structural features that control Pt/DNA interactions and the anticancer activity of Pt drugs are described. [$^1$H,$^{15}$N]-HSQC 2D NMR spectroscopic studies of the reactions of cisplatin with oligonucleotides containing ApG and GpA binding sites reveal dramatic differences in the rates of formation of monofunctional adducts at the two sites. When the reactant is *cis*-$[Pt(NH_3)_2(OH_2)_2]^{2+}$ no such differences are observed suggesting that outer-sphere interactions between the reactant and the oligonucleotide may play a substantial role in determining the rates. Rates of closure to the bifunctional adducts are similar to those observed for cisplatin. Studies of the adduct profiles formed by sterically bulky and/or optically active complexes reveal that steric interactions play a major role in mediating the binding of Pt(II) to DNA but that hydrogen bonds play less of a role. *In vitro* cytotoxic activities for these complexes do not always follow the trends that would be expected on the basis of the adduct profiles.

### 1. Introduction

Although it has been widely accepted that the binding of Pt(II) to DNA is ultimately responsible for the anticancer activity of Pt based anticancer drugs [1-4], the factors that control the DNA binding and how these influence the anticancer activity are poorly understood [5]. For example, molecular models and some crystal structures reveal hydrogen bonds between the oxygen atoms of DNA and the amine ligands that remain bound to Pt [6-13]. On the basis of these observations it has been proposed that the ability to form such hydrogen bonds is required for DNA binding and anticancer activity [9,14]. This suggestion is consistent with the observation that replacement of all H atoms on the amine groups with alkyl groups greatly diminishes the anticancer activity [15] but is not consistent with the increasing number of compounds that are active but do not have H(amine) atoms. An alternative explanation is that it is the small size of the H(amine) atoms rather than their hydrogen bonding ability that is important [5,16] but the role of steric factors in mediating Pt/DNA interactions has received relatively little attention.

To date, most studies of Pt/DNA interactions have focused on the final products but to gain an understanding of what controls the product distribution each step needs to be examined. The overwhelmingly preferred binding sites are the GpG and ApG sequences [17-21] and there are indications that sequences with more than two guanine bases are favoured [22,23]. Binding to isolated guanine bases is inherently preferred over binding to isolated adenine bases [24] but outer-sphere complex formation prior to binding also has the potential to influence the final product distribution. If this is the case then the bases flanking a binding site could control binding and ultimately determine the effectiveness of the drug in terms of its ability to bind to a particular sequence and produce an anticancer effect.

In the present paper we summarise the results of our recent studies of the factors that control Pt/DNA interactions. In particular we have investigated the reasons for the preference of drugs such as cisplatin (*cis*-diamminedichloroplatinum(II), *cis*-$[Pt(NH_3)_2Cl_2]$) to bind to GpG and ApG sequences and their failure to bind bifunctionally to GpA sequences of DNA. We have also addressed the roles of hydrogen bonding and steric interactions in influencing the binding of Pt complexes to DNA and influencing the anticancer activity of these complexes.

### 2. Results and Discussion

### *2.1 Kinetic Studies of Pt Binding to GpG, ApG and GpA Sequences*

The binding of even simple complexes such as cisplatin to bispurine sequences of DNA (GpG, ApG or GpA) is a highly complicated process. Hydrolysis of one of the chloro ligands is followed by formation of one or two monofunctional adducts and each of these will then close at different rates to produce the bifunctional rates. Thus, a minimum of five species can be anticipated, as shown in Scheme 1 for the reaction at an ApG site, and following the appearance and disappearance of these species is difficult. Chottard and colleagues have successfully used HPLC techniques to quantify trapped intermediates in the reaction between *cis*-$[Pt(NH_3)_2(OH_2)_2]^{2+}$ and a variety of oligonucleotides containing the GpG sequence [25,26]. They found little selectivity for either the 5'-G or the 3'-G in the formation of the monofunctional adducts but found substantially more rapid closure to the bifunctional adduct from the 3'-G monofunctional. Sadler, Berners-Price and colleagues have obtained similar results using [$^1$H,$^{15}$N]-HSQC 2D NMR spectroscopy [27]. They also studied the reaction of cisplatin with oligonucleotides and found a higher rate of binding to the 3' guanine of a GpG pair and faster closure to the bifunctional adduct from this base [28,29].

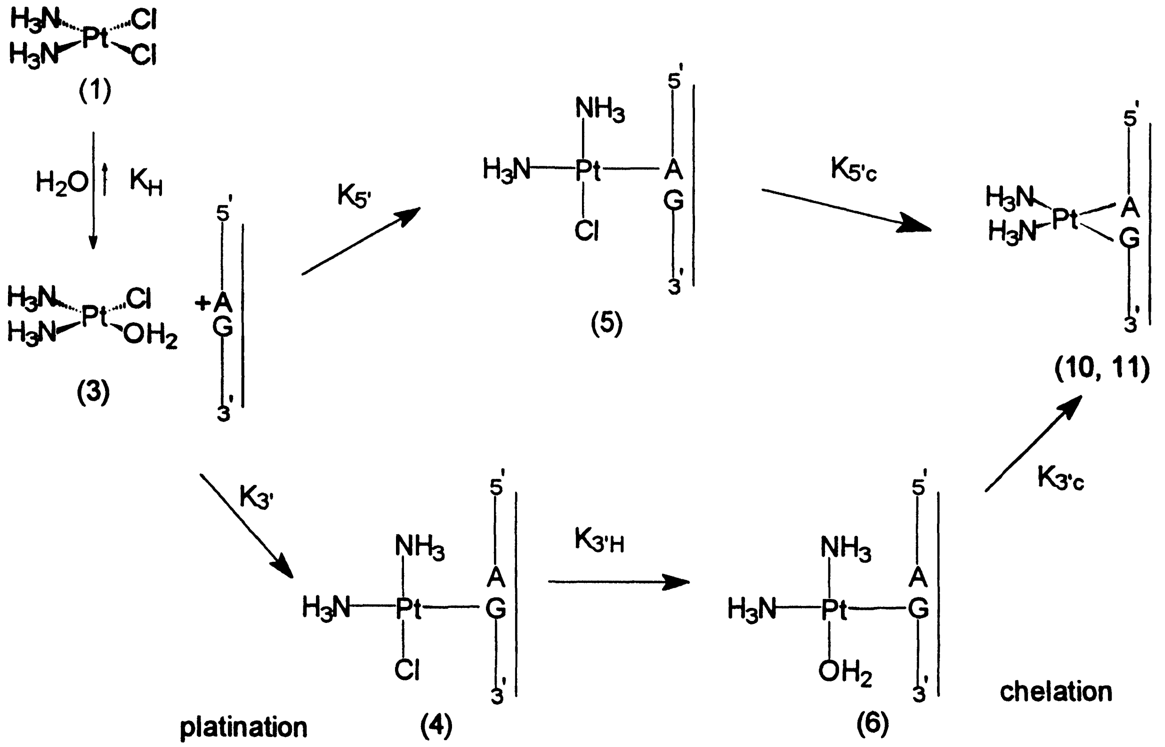

**Scheme I**

We have used [$^1$H,$^{15}$N]-HSQC 2D NMR spectroscopy to follow the rates of binding of cisplatin and *cis*-$[Pt(NH_3)_2(OH_2)_2]^{2+}$ to the self-complementary sequences d(5¥-AATT**AG**TACTAATT-3¥) and d(5¥-AATT**GA**TATCAATT-3¥) containing ApG and GpA sites respectively [30]. A typical spectrum showing starting materials, intermediates and the final product for reaction at the ApG site is shown in Figure 1 and a plot showing the appearance and disappearance of each of these species is shown in Figure 2. Similar plots were obtained for the reaction at the GpA site. Rate constants for each step were extracted from numerically optimised fits to these data curves and the calculated curves are also shown in Figure 2. Calculated rate constants are listed in Table I along with those reported previously for reaction at the GpG site [28,29].

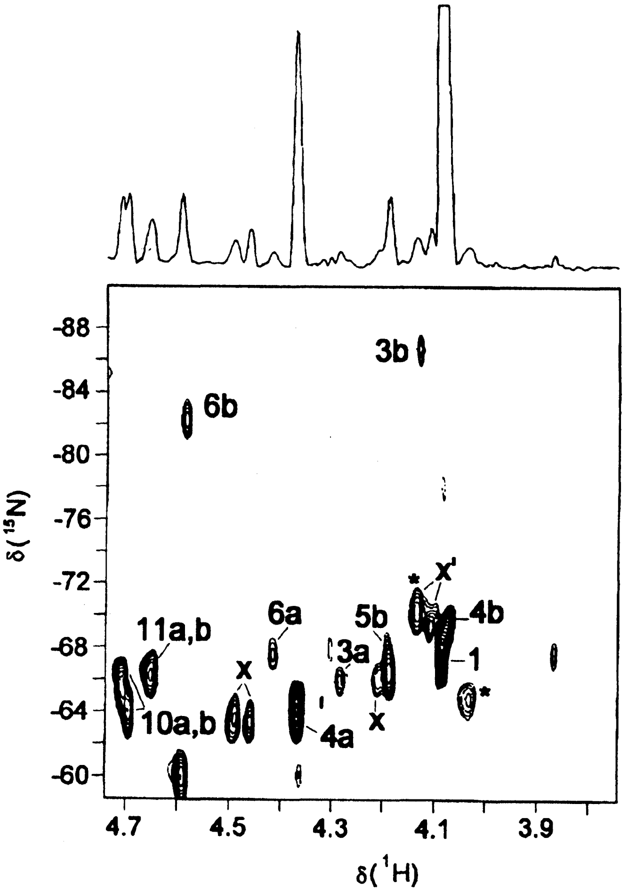

**Figure 1.** [$^1$H,$^{15}$N]-HSQC 2D NMR spectrum from the reaction between cisplatin and the oligonucleotide d(5¥-AATT**AG**TACTAATT-3¥) taken from reference 30. Numbered species are defined in Scheme 1.

Rates of monofunctional adduct formation at the ApG site are broadly similar to those at the GpG site. Binding at the 3'-G is more rapid than binding to the 3'-G of the GpG sequence and binding at the 5'-A is remarkably similar in rate to binding at the 5'-G of GpG. In contrast rates of monofunctional binding to GpA site are markedly slower. Thus, binding at the 3'-A is two orders of magnitude slower than binding to the 3'-G's of GpG and ApG and binding at the 5'-G is nearly an order of magnitude slower than is binding to the 3'-bases of GpG and ApG. Some tentative conclusions can be drawn from these results; (i) the normal preference for binding to guanine over adenine can be substantially affected by both flanking sequences and the DNA structure, (ii) the base on the 3' side of a binding site exerts a substantially greater influence on the rate of binding than does the base on the 5' side and (iii) the rates of monofunctional adduct formation are an important determinant of the final adduct profile.

Rates of closure to the bifunctional adduct are remarkably similar for all three sequences and follow the same trends. Thus closure from the 3' side is faster, ranging from 1.6 to 3.2 x $10^{-5}$ $s^{-1}$, than closure from the 5' side that ranges from 0.18 to 0.24 x $10^{-5}$ $s^{-1}$. The constancy of these rates is despite the fact that the bases are guanine in some cases and adenine in others providing further evidence that the DNA structure can overrule the inherent preference for binding to guanine over adenine. They also show that the major reason for the non-formation of the bifunctional GpA adduct is the slow rate of formation of the monofunctional adduct, though this is further exacerbated by the fact that the more rapidly formed monofunctional adduct at the GpA sequence is the one that closes more slowly to the bifunctional adduct.

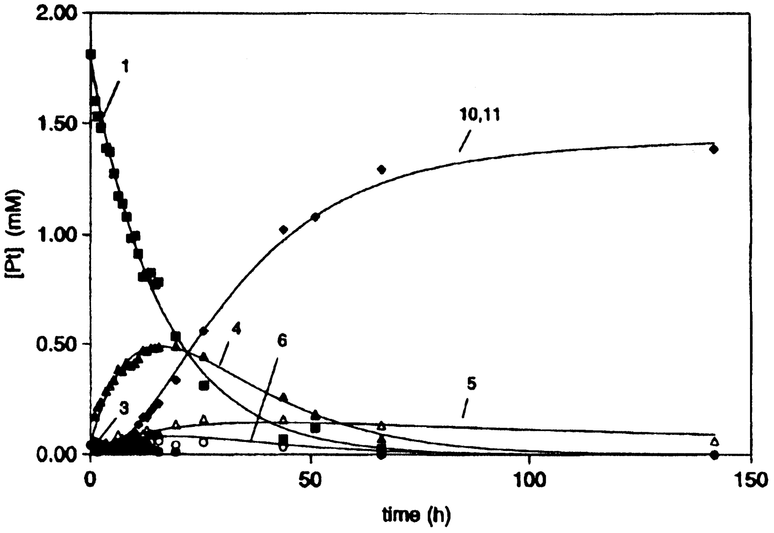

**Figure 2.** Reaction profile for the reaction between cisplatin and the oligonucleotide d(5¥-AATT**AG**TACTAATT-3¥) taken from reference 30.

**Table 1.** Rate constants for the reactions between cisplatin and oligonucleotides

| Rate Constant | AG[30] | GA[30] | GG[28] |
|---|---|---|---|
| $K_H$ ($10^{-5}$ $s^{-1}$) | 1.71(0.03) | 1.13(0.02) | 1.83(0.03) |
| $K_3$ ($M^{-1}$ $s^{-1}$) | 0.96(0.07) | 0.005(0.001) | 0.47(0.04) |
| $K_5$ ($M^{-1}$ $s^{-1}$) | 0.14(0.02) | 0.023(0.002) | 0.15(0.03) |
| $K_{3c}$ ($10^{-5}$ $s^{-1}$) | 1.59(0.05) | 2.1(0.5) | 3.2(0.1) |
| $K_{5c}$ ($10^{-5}$ $s^{-1}$) | 0.18(0.07) | 0.20(0.02) | 0.24(0.18) |

**Table 2.** Rate Constants for the Reactions Between *cis*-$[Pt(NH_3)_2(OH_2)_2]^{2+}$ and DNA

| Rate Constant | AG[30] | GA[30] | GG[27] |
|---|---|---|---|
| $K_3$ ($M^{-1}$ $s^{-1}$) | 0.419(0.009) | not observed | too fast to measure |
| $K_5$ ($M^{-1}$ $s^{-1}$) | not observed | 0.500(0.007) | too fast to measure |
| $K_{3c}$ ($10^{-5}$ $s^{-1}$) | 3.71(0.05) | not observed | 25(3) |
| $K_{5c}$ ($10^{-5}$ $s^{-1}$) | not observed | 0.162(0.005) | 4.9(0.4) |

Rates of the reactions of *cis*-$[Pt(NH_3)_2(OH_2)_2]^{2+}$ at the same three sequences (Table 2) are different in many respects to those for cisplatin. Only G monofunctional adducts are observed in the reactions of *cis*-$[Pt(NH_3)_2(OH_2)_2]^{2+}$ at both the ApG and GpA sites and these form at similar rates. Thus, the rate of binding at GpA in particular is strongly influenced by the nature of the incoming reactant consistent with outer-sphere complex formation having a strong influence on the reaction rate and possibly on sequence selectivity. The rates of closure to the bifunctional adducts are similar to those observed for cisplatin and because the 5'-G monofunctional adduct formed at the GpA site is the one that is slow to close, the overall rate of formation of the GpA bifunctional adduct is much slower than formation of the ApG or GpG adducts. Curiously, the rates of closure were approximately an order of magnitude faster at the GpG sequence and the rates of monofunctional adduct formation were too rapid to measure.

*2.2 Stereoselective Interactions Between Pt and DNA*

The relative importance of interstrand and intrastrand bifunctional adducts remains an open question and we have previously described the design of a series of compounds to probe this question [31-33].

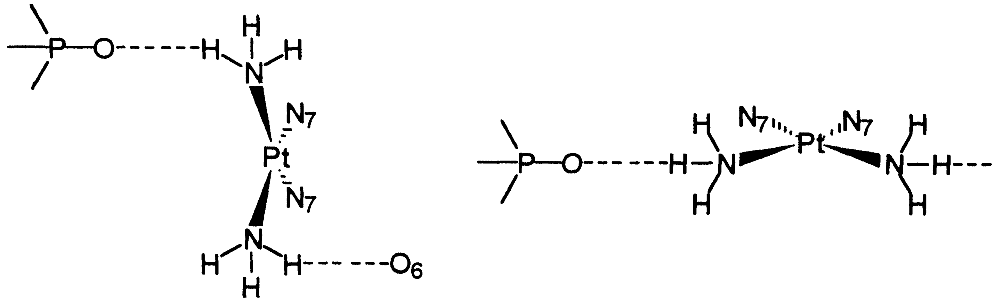

**Intrastrand GpG crosslink** **Interstrand GG crosslink**

**Scheme II**

The design was based on the differences in the orientations of the hydrogen bonds formed in each type of adduct and therefore the compounds were also potential probes of the importance of these hydrogen bonds. In the interstrand adduct the hydrogen bonds lie approximately in the coordination plane while in the intrastrand adduct they are approximately perpendicular to the coordination plane as shown in Scheme II.

Thus, compounds that are only able to form hydrogen bonds close to the coordination plane are expected to be more suited to forming interstrand adducts. The complex $[PtCl_2(hpip)]$ (hpip = 1,4-diazacycloheptane, Scheme III) was the first selected on this basis and we were able to show that it formed interstrand adducts as readily as cisplatin and also that its ability to form intrastrand GpG adducts was substantially diminished compared to that of cisplatin [34]. Taken with the unusually low *in vitro* cytotoxicity of $[PtCl_2(hpip)]$, these results may indicate that the interstrand adducts do not contribute significantly to the anti-cancer activity of Pt(II) based anticancer drugs [34]. This is a question that needs further investigation and we are currently preparing and testing compounds related to $[PtCl_2(hpip)]$.

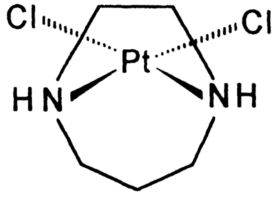

**Scheme III**

When measuring the intrastrand adducts formed by $[PtCl_2(hpip)]$ on DNA two GpG adducts in substantially different proportions are observed [34]. The same two peaks are observed in the reaction of $[PtCl_2(hpip)]$ with isolated GpG, this time in equal proportions [35]. 1D and 2D NMR spectra of these two peaks showed them to be isomers of the expected [Pt(GpG)(hpip)] complex [35]. Two isomers are expected because of the asymmetry of the hpip and GpG ligands. Close contacts between the H8 atoms of the GpG and protons on either the ethylene or propylene links on the backbone of the hpip ligands were evident in the NOESY spectra and these allowed us to identify the isomers [35]. Peak I corresponds to the isomer in which the propylene link is adjacent to the floor of the major groove of the DNA and peak II

to the isomer in which the ethylene link adopts this position. Molecular mechanics models of each of these isomers on a DNA fragment show substantial differences in the severity of the steric clashes between the linking groups and the DNA [36]. Thus, in isomer I close contacts occur between in the propylene link and the DNA. These produce substantial distortions in the coordination geometry about the Pt and contacts as short as 2.55 ≈ remain in the energy-minimised models. In isomer II the contacts are significantly longer (>2.72 ≈) and the distortion in the coordination sphere less. These observations are in accord with the proportions of each isomer that are observed with isomer I only accounting for 7% of the Pt bound to the DNA and isomer II accounting for 25%. The amount of isomer II formed is close to that expected on the basis of the results for cisplatin, assuming that the ability to form two isomers leads to a 50% reduction in the amount of each expected. Thus, these results are all consistent with steric interactions between the amine ligand and the DNA having a significant influence on the interactions with the DNA and on the final adduct profile. In neither isomer are hydrogen bonds observed between the H(amine) atoms and the DNA but, as mentioned, the amount of isomer II formed is not greatly diminished compared to cisplatin. Thus, the ability to form these hydrogen bonds does not appear to be a major mediator of Pt/DNA interactions in this case.

*2.3 Enantioselective Complexes as Probes of the Roles of Steric and Hydrogen Bonding Interactions*

In order to further test the importance of hydrogen bonds between the amines and DNA in mediating the formation of GpG intrastrand adducts we designed and prepared a series of complexes of chiral diamine ligands. The design principle is shown in Scheme IV and is based on the orientation of the hydrogen bonds and the chirality of the GpG adduct. Enantiomer A has two amine protons disposed appropriately for the formation of the hydrogen bonds while enantiomer B has alkyl groups disposed toward the hydrogen bond acceptors. If these hydrogen bonds are important then enantiomer A should be readily able to form the GpG adduct and enantiomer B less so. Additionally, if the GpG adduct is primarily responsible for the anticancer activity of such complexes then enantiomer A should be substantially more active than enantiomer B.

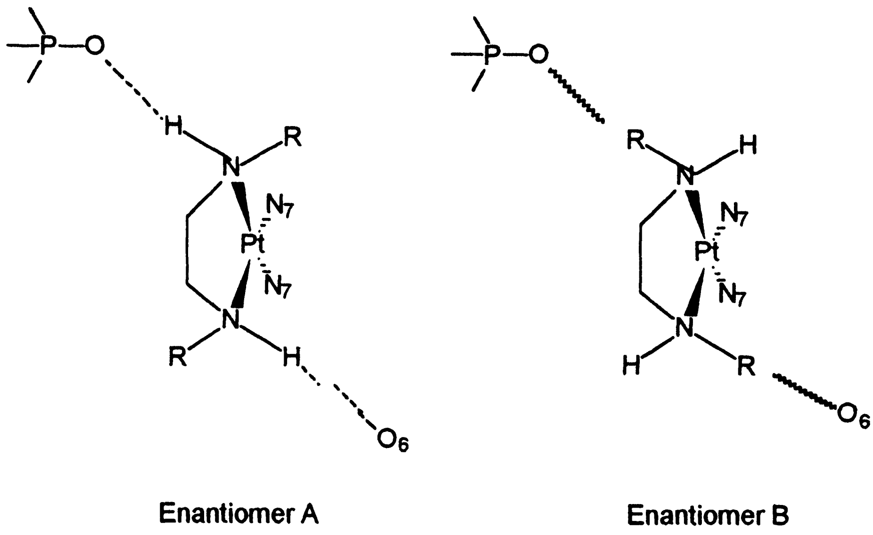

**Scheme IV**

Obtaining compounds that meet these design goals has proven difficult. Chirality at the amine groups is unstable and therefore ligands with inert chiral centres adjacent to the amine were chosen in the expectation that they would impose the required chirality via atropisomerism. Our first target compounds, [$PtCl_2$(2,4-pentanediamine)] and [$PtCl_2$(*N*,*N*'-dimethyl-1,2-cyclohexanediamine)], did not meet these requirements because two diastereomers were observed in each case [31]. However, variants of the ahaz ligand (ahaz =

3-aminohexahydroazepine, Scheme V), were successful in that the undesired diastereomer accounted for less than 5% of the complexes [33]. The endocyclic nature of one of the amine groups means the chirality at that centre is rigorously imposed and an alkyl group on the exocyclic amine adopts the orientation that gives rise to the required *trans* hydrogen atoms more than 95% of the time.

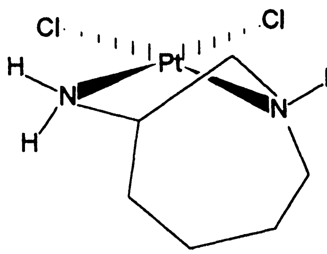

**Scheme V**

In the first phase of this study we studied the enantiomers of the parent complex [$PtCl_2$(ahaz)]. As with [$PtCl_2$(hpip)], the inequivalence of the amine groups of the ahaz ligand means that two GpG adducts are observed for each enantiomer as shown in Scheme VI. The amounts of each of these formed, measured by enzymatic digestion and HPLC separation, are also shown in Scheme VI.

For the *R*-enantiomer the two isomers are observed in approximately equal amounts but for the *S*-enantiomer the amounts differ substantially, one being similar to the *R* enantiomer and the other more that three times as abundant. Using 2D-NMR spectroscopy were have established that the most abundant adduct is the one in which the exocyclic $NH_2$ group of the *S*-enantiomer is oriented toward the 5' side of the adduct (*S*(a) in Scheme VI). Molecular mechanics models of the four isomers provided a possible explanation for these observations because for *R*(a), *R*(b) and *S*(b) substantial steric clashes are observed between the ligands and the DNA but for *S*(a) the entire ligand lies in the major groove and makes no close contacts with the DNA. Thus, these results provide further support for the notion that steric clashes between the complex and DNA can exert a significant influence on the adduct profile. They also provide evidence that the ability to form hydrogen bonds may not be as important because for each of the *R* isomers only one of the hydrogen bonds is able to form whereas for each of the *S* isomers both hydrogen bonds can form but the amounts of each isomer formed do not correlate with the number of hydrogen bonds that can form.

The *S*-enantiomer forms substantially more bifunctional GpG adducts (37%) than does the *R*-enantiomer (21%). Thus, if this adduct was responsible for effecting the anti-cancer activity, then the *S*-enantiomer would be expected to be more active. We have measured the activity of the both enantiomers against a number of cell lines and the results are given in Table III. In nearly all cases the two enantiomers have similar activities and in some cases the *R*-enantiomer appears to be the more active, in contrast to the expectations outlined above. Other factors such as cellular uptake could contribute to these results but we have shown that there is no enantioselective difference in the uptake [32]. Alternatively, it has been suggested that anti-cancer activity depends on the ability of proteins to recognise adducts and initiate events that lead to cell death [37]. It may be that the additional steric interactions observed for the *R*-enantiomer lead to additional bending of the DNA that in turn lead to better recognition.

*In vitro* cytotoxicities of complexes of the substituted ahaz complexes (Scheme VII) are given in Table IV [33]. Addition of a methyl or ethyl group to the exocyclic amine results in a nearly five-fold drop in the activity of the complexes. This is in contradiction to the structure-activity rule that high activity is usually observed when each amine group has one or more hydrogen atoms. When two methyl groups are added to the exocyclic nitrogen the activity does not change significantly compared to the value for a single added methyl group. Again, this is unexpected because structure-activity rules suggest that removal of both hydrogen atoms from one of the amine groups should lead to a substantial drop in the cytotoxicity

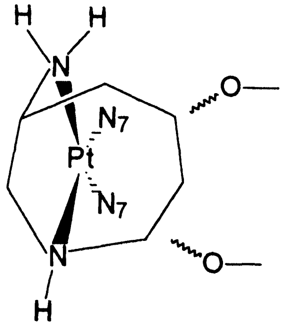

(a) 9 or 12%

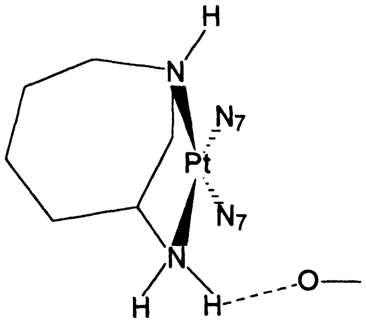

(b) 12 or 9%

*R*-ahaz

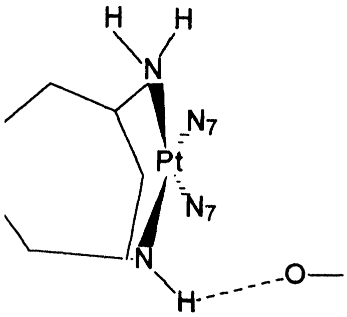

(a) 30%

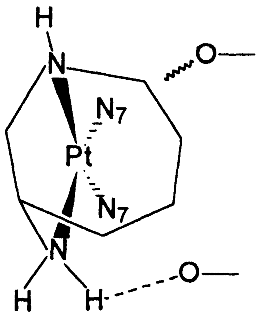

(b) 7%

*S*-ahaz

**Scheme VI**

**Table 3.** Cytotoxicity ($IC_{50}$) values (μM) determined in human bladder tumour cell lines. Numbers in parentheses are standard errors in units of the last significant figure quoted.

| platinum complexes | $IC_{50}$ (μM) | | | |
|---|---|---|---|---|
| | UCRU BL13/0 | PC9 | PC9cisR | DU145 |
| MTT assay | | | | |
| cisplatin | 1.9(5) | 1.4(2) | 11.7(31) | 1.8(8) |
| [Pt(*R*-ahaz)$Cl_2$] | 1.3(2) | 5.9(1) | 16.0(10) | 2.9(1) |
| [Pt(*S*-ahaz)$Cl_2$] | 2.0(2) | 3.0(4) | 15.5(13) | 3.4(3) |
| SRB assay | | | | |
| cisplatin | 9.5(18) | 5.0(5) | >17.5 | 1.0(2) |
| [Pt(*R*-ahaz)$Cl_2$] | 7.2(4) | 11.0(15) | 27.3(35) | 3.3(2) |
| [Pt(*S*-ahaz)$Cl_2$] | 12.3(7) | 11.7(9) | 25.0(15) | 3.7(3) |

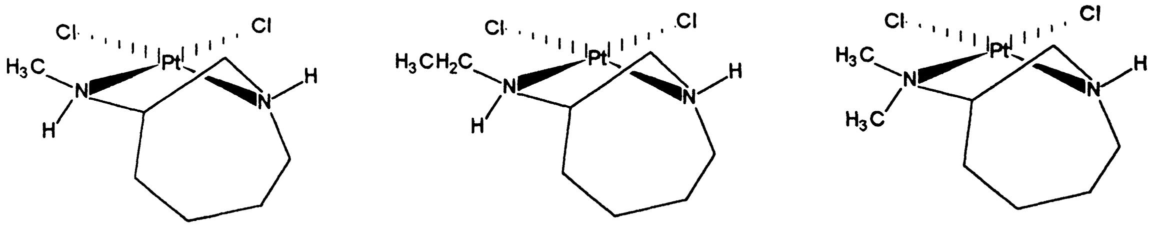

$[PtCl_2(meahaz)]$ $[PtCl_2(etahaz)]$ $[PtCl_2(dimeahaz)]$

**Scheme VII**

In all of these complexes and against most cell lines the *R*-enantiomer is significantly more active than the *S*. Thus, it is clear from the foregoing studies that much remains to be established about what controls Pt/DNA interactions and how these in turn relate to anti-cancer activity.

**Table 4.** $IC_{50}$ values.

| | **BL13/0** | **PC9** | **PC9-cisR** | **DU145** |
|---|---|---|---|---|
| **Drug** | mean | mean | mean | mean |
| *cisplatin* | 1.9(0.5) | 1.4(0.2) | 11.7(3.1) | 1.8(0.8) |
| [Pt(*R*-ahaz)$Cl_2$] | 1. 3(0.2) | 5.9(0.1) | 16.0(1.0) | 2.9(0.1) |
| [Pt(*S*-ahaz)$Cl_2$] | 2.0(0.2) | 3.0(0.1) | 15.3(1.3) | 3.4(0.3) |
| [Pt(*R*-meahaz)$Cl_2$] | 7.3(1.3) | 13.3(1.9) | 19.0(1.2) | 6.7(1.3) |
| [Pt(*S*-meahaz)$Cl_2$] | 11.7(1.6) | 13.7(1.2) | 38.3(8.0) | 10.1(1.7) |
| [Pt(*R*-etahaz)$Cl_2$] | 7.4(1.1) | 20(3.5) | 44(4.4) | 9.5(0.9) |
| [Pt(*S*-etahaz)$Cl_2$] | 19(2.1) | 31(4) | 79.7 (8.5) | 21.3(2.4) |
| [Pt(*R*-dimeahaz)$Cl_2$] | 7.7(2.1) | 13.5(3.4) | 29.3(1.5) | 7.3(0.2) |
| [Pt(*S*-dimeahaz)$Cl_2$] | 10.2(1.4) | 11.8(2.6) | 28.7(2.2) | 11.5(0.8) |

**Acknowledgments**
We wish to thank the Australian Research Council and the Sydney University Cancer Research Fund for financial support.

**References**
1. A.L. Pinto and S.J.Lippard, *Biochem. Biophys. Acta*, **780** (1985) 167.
2. J.L. van der Veer and J. Reedijk, *Chemistry in Britain*, **24** (1988) 775.
3. N. Sheibani, M.M. Jennerwein and A. Eastman, *Biochemistry*, **28** (1989) 3120.
4. J. Reedijk, *J. Chem. Soc., Chem. Commun.* (1996) 801.
5. T.W. Hambley, *Coord. Chem. Rev.*, **166** (1997) 181.
6. J. Kozelka, G.A. Pestko, S.J. Lippard and G.J. Quigley, *J. Am. Chem. Soc.*, **107** (1985) 4079.
7. J. Kozelka, G.A. Petsko, G.J. Quigley and S.J. Lippard, *Inorg. Chem.*, **25** (1986) 1077
8. J. Kozelka, S. Archer, G.A. Petsko, S.J. Lippard and G.J. Quigley, *Biopolymers*, **26** (1987) 1245.
9. T.W. Hambley, *Inorg. Chem.*, **30** (1991) 937.
10. T.W. Hambley, *Inorg. Chem.*, **27** (1988) 1073.
11. S.E. Sherman, D. Gibson, A.H.-J. Wang and S.J. Lippard, *Science*, **230** (1985) 412.
12. P.M. Takahara, A.C. Rosenzweig, C.A. Frederick and S.J. Lippard, *Nature*, **377** (1995) 649.

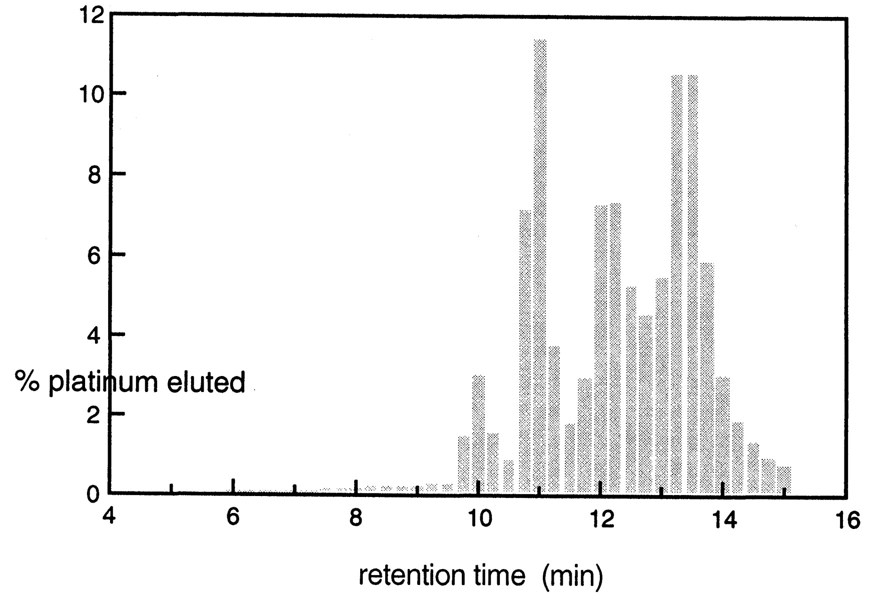

13. P.M. Takahara, C.A. Frederick and S.J. Lippard, *J. Am. Chem. Soc.*, **118** (1996) 12309.
14. J. Reedijk, *Inorg. Chim. Acta*, **198-200** (1992) 873.
15. M.J. Cleare and J.D. Hoeschele, *Bioinorg. Chem.*, **2** (1973) 187.
16. T.W. Hambley, *Comm. Inorg. Chem.*, **14** (1992) 1.
17. A.M.J. Fichtinger-Schepman, P.H.M. Lohman and J. Reedijk, *Nucl. Acids Res.*, **10** (1982) 5345.
18. A.M.J. Fichtinger-Schepman, J.L. van der Veer, J.H.J. den Hartog, P.H.M. Lohman and J. Reedijk, *Biochemistry*, **24** (1985) 707.
19. A. Eastman, *Biochemistry*, **22** (1983) 3927.
20. A. Eastman, *Biochemistry*, **24** (1985) 5027.
21. A. Eastman, *Biochemistry*, **25** (1986) 3912.
22. G.L. Cohen, J.A. Ledner, W.R. Bauer, H.M. Ushay, C. Caravana and S.J. Lippard, *J. Am. Chem. Soc.*, **102** (1980) 2487.
23. T.D. Tullius, S.J. Lippard, *Proc. Natl. Acad. Sci. USA*, **79** (1982) 3489.
24. R.B. Martin, *Acc. Chem. Res.*, **18** (1985) 32.
25. F. Gonnet, F. Reeder, J. Kozelka and J.-C. Chottard, *Inorg. Chem.*, **35** (1996) 1653.
26. F. Reeder, F. Gonnet, J. Kozelka, and J.-C. Chottard, *Chem. Eur. J.*, **2** (1996) 1068.
27. F. Reeder, Z. Guo, P. del Socorro Murdoch, A. Corazza, T.W. Hambley, S.J. Berners-Price, J.-C. Chottard and P.J. Sadler, *Eur. J. Biochem.*, **249** (1997) 370.
28. K.J. Barnham, S.J. Berners-Price, T.A. Frenkiel, U. Frey, P.J. Sadler, *Angew. Chem. Int. Edn. Engl.*, **34** (1995) 1874.
29. S.J. Berners-Price, K.J. Barnham, U. Frey, P.J. Sadler, *Chem. Eur. J.*, **2** (1996) 1283.
30. M.S. Davies, S.J. Berners-Price and T.W. Hambley, submitted.
31. K. Vickery, A. M. Bonin, R. R. Fenton, S. O'Mara, P.J. Russell, L.K. Webster and T.W. Hambley, *J. Med. Chem.*, **36** (1993) 3663.
32. R.R. Fenton, W.J. Esdale, H.M. Er, S.M. O'Mara, M.J. McKeage, P.M. Russell, and T.W. Hambley *J. Med. Chem.,* **40** (1997) 1090.
33. E.M. Rezler, R.R. Fenton, W.J. Esdale, M.J. McKeage, P.J. Russell, and T.W. Hambley *J. Med. Chem.,* **40** (1997) 3508.
34. E.C.H. Ling, G.W. Allen and T.W. Hambley, *J. Am. Chem. Soc.*, **116** (1994) 2673.
35. T.W. Hambley, E.C.H. Ling and B.A. Messerle, *Inorg. Chem.*, **35** (1996) 4663.
36. T.W. Hambley and E.C.H. Ling, *Inorg. Chem.*, submitted.
37. P.M. Pil and S.J. Lippard, *Science*, **256** (1992) 234.

**Received: June 30, 1998 - Accepted: July 14, 1998**

